# COVID-19 Related Distress Is Associated With Alcohol Problems, Social Media and Food Addiction Symptoms: Insights From the Italian Experience During the Lockdown

**DOI:** 10.3389/fpsyt.2020.577135

**Published:** 2020-11-25

**Authors:** Angelo Panno, Giuseppe Alessio Carbone, Chiara Massullo, Benedetto Farina, Claudio Imperatori

**Affiliations:** Cognitive and Clinical Psychology Laboratory, Department of Human Science, European University of Rome, Rome, Italy

**Keywords:** COVID-19 related distress, problematic alcohol use, social media addiction, food addiction, impulsivity, lockdown

## Abstract

**Background:** Several scholars hypothesize that one of the most negative impacts of the coronavirus disease 2019 (COVID-19) crisis would concern the increase of prevalence and severity of both substances and behavioral addiction. Despite the general concerns about the increase of prevalence and severity of addictions related to the COVID-19 emergency, few data are still available. Thus, the main aim of this study was to investigate the association between COVID-19 related distress and: (i) alcohol problems, (ii) social media addiction (SMA) symptoms, (iii) food addiction (FA) symptoms.

**Methods:** A national online-survey was carried out during the Italian lockdown (i.e., 9 March 2020–4 May 2020). In the current study, 1,519 participants (365 men and 1,154 women, mean age: 28.49 ± 10.89 years) were included. The survey included socio-demographic related items (e.g., age, sex, residential regions, education level, civil status, tobacco use, etc.), as well as *ad-hoc* developed questions aimed to investigate COVID-19 related variables (e.g., isolation/quarantine, personal diagnosis to COVID-19, friends or relatives with COVID-19 diagnosis, etc.). Participants also completed the following self-report measures in order to investigate: the psychological impact of COVID-19, alcohol problems, SMA symptoms, FA symptoms, and impulsivity.

**Results:** The psychological impact of COVID-19 was independently associated with alcohol problems (β = 0.058, *p* = 0.043), SMA symptoms (β = 0.259, *p* < 0.001), and FA symptoms (β = 0.150, *p* < 0.001).

**Conclusion:** Taken together, our results seem to confirm the general concerns about the negative impacts of the COVID-19 emergency on addictive behaviors, suggesting that this issue should be carefully monitored.

## Introduction

The coronavirus disease 2019 (COVID-19) outbreak is a global health crisis currently (i.e., 05th November 2020) involving 190 nations with more than 48,450,000 confirmed cases and over 1,220,000 deaths around the world (1). This emergency is radically affecting our everyday life with serious consequences from the economic, health and psychosocial perspectives. The sudden development of the epidemic makes it necessary for timely research data to inform clinicians' interventions and policy-makers' decisional processes.

The adverse impacts of the COVID-19 outbreak on mental health concern are not only relevant to frontline staff working in a high-stress environment (2, 3) but also to millions of people forced into isolation (4, 5). A recent meta-analysis on 33,062 healthcare workers reported a prevalence of 22.8% for depression, 23.2% for anxiety symptoms and 38.9% for insomnia during the COVID-19 outbreak (6). Similarly, in response to the problems posed by the pandemic, the lockdown public health strategy, reducing access to family, friends, and other social support systems, produced a general worsening of psychosocial well-being (7–11).

Scholars hypothesize that one of the most negative impacts of the COVID-19 emergency is concerned with the increased of prevalence and severity of both substance and behavioral addictions (11–15). It is well-known that individuals who are isolated and stressed, as well as much of the population during the COVID-19 emergency, frequently turn to substances or rewarded behaviors/actions (e.g., online gaming) to cope with their negative feelings (11, 12). It has been proposed (11, 16) that staying indoors for long periods may increase the risk of compulsive overeating consumption of high calorie food (i.e., foods with high sugar and/or fat), a specific clinical condition known as Food Addiction [FA; (17–20)]. Although not formally recognized in the last edition of the Diagnostic and Statistical Manual of Mental Disorders [DSM-5; (21)], several studies have shown strong biological (i.e., altered dopamine expression) and behavioral overlaps (e.g., compulsive overeating in stressful situations) between drug use and uncontrolled consumption of hyper-palatable foods (17, 18, 22).

Despite the general concerns about the increase of prevalence and severity of addictions related to the COVID-19 emergency, few data are yet available (14). For example, a prospective cohort study of 1,442 health profession students showed that internet addiction severity was associated with outbreak-related psychological distress and symptoms of acute stress reaction (23). Empirical data aimed at providing the greatest amount of information to cope with emergency situations triggered by phenomena, such as the COVID-19 pandemic are needed. A main aim of these kind of studies (e.g., large surveys at national level or longitudinal research designs) consists of shedding light on these phenomena in order to provide useful public health information that might be taken into account by policy-makers and health professionals when such emergency situations occur. On the one hand, clinicians need such information to tailor applied interventions. On the other hand, policy-makers have to take into account such information to support and stimulate clinical interventions, as well as to develop efficient policies aimed at addressing issues of public health.

The main aim of this study was to investigate the association between COVID-19 related stress and the severity of some addictions during the Italian lockdown (9 March 2020–4 May 2020), one of the first countries most affected by the pandemic. We focused on three specific types of addiction that do not entail the intake of substances considered illegal in the nation of interest for the study, because such addictions could be affected by restrictions due to lockdown (e.g., inability to leave the house for no proven reasons). More specifically, we focused on kinds of reinforcing stimuli easily available at home during the lockdown: alcohol, social media and food. In line with some reports (11–15), we hypothesized that COVID-19 related distress would be positively and independently associated with: (i) alcohol problems, (ii) social media addiction (SMA) symptoms, (iii) FA symptoms (when controlling for potential confounding variables that have been traditionally associated with addictions).

## Materials and Methods

### Participants

Data here reported were part of a wider project designed to investigate the psychopathological impacts of the COVID-19. Participants completed an anonymous online survey, after reading and signed a written informed consent. The survey link, preceded by a brief description of the study aim (i.e., understanding the impact of the COVID-19 pandemic on mental health) was shared through social media (e.g., Facebook, WhatsApp, LinkedIn, Instagram), mailing lists, and personal contacts, from 30th March to 4th May 2020 (i.e., “phase one” of the pandemic emergency in Italy where the exponential curve was growing). Participants could complete the survey directly from their smartphone, tablet, or computer.

All Italian regions have been involved in the study. All participants voluntarily took the survey (i.e., they did not receive payment or compensation). This research was approved by the ethics committee of the European University of Rome (Prot. N.004/20) in line with the Helsinki declaration standards. Inclusion criteria were: (i) being resident in Italy during the lockdown, (ii) age ≥ 18 years, (iii) correct response to an item of attentional quality check (i.e., responding to this question “completely agree” or skip the question). The exclusion criteria were: (i) the inability to understand written Italian, and (ii) the refusal to provide written consent. The online survey was completed by 1,765 participants: 35 were excluded because they were not Italian resident, and 211 were excluded because they failed to respond to the attentional quality check item. The final sample consisted in 1,519 participants (365 men and 1,154 women, mean age: 28.49 ± 10.89 years; range 18–74). We performed a *priori* power analysis through G^*^Power 3.1 software (24). It indicated that, given a probability level of 0.05, a sample size of 1,100 was required to provide a satisfactory statistical power (1– β= 95%) to also identify a potential small effect size (*f* ^2^= 0.02) in a two-sided test with 7 tested predictors and 17 total number of predictors.

### Materials

The survey included socio-demographic items (e.g., age, sex, residential region, education level, etc.), as well as *ad-hoc* developed questions aimed at investigating COVID-19 related variables (e.g., isolation/quarantine, personal diagnosis to COVID-19, friends or relatives with COVID-19 diagnosis, etc.). Participants were also instructed (25) to measure and accurately report their current height and weight to calculate body mass index (BMI). Based on data provided by Italian Ministry of Health (26), we assessed the number of COVID-19 infected people in the region of interest. In particular, we recorded the number of infected referred to the day before the compilation of the survey, in the specific regions where the participant lived during the lockdown. Every day at 06.00 p.m., the newscast informed the population of the number of infected people. We consider these data to estimate the psychological pressure related to the number of infected in the region of interest. There was a wide difference concerning the number of infected across the regions, thus this index provided a measure to estimate people's perceived pressure due to the spread of the COVID-19's infection. Participants also completed the following self-report measures to investigate: COVID-19 related distress, alcohol problems, SMA symptoms, FA symptoms and impulsivity. All variables considered in the study are reported in Table 1.

**Table 1:** Descriptive statistics for the sample (*N* = 1519).

**Variables**	
Women–*N* (%)	1,154 (76.0)
Age–M ± SD	28.49 ± 10.89
North Italy–*N* (%)	521 (34.3)
Central Italy–*N* (%)	639 (42.1)
South Italy–*N* (%)	359 (23.6)
Self-reported BMI–M ± SD	23.28 ± 4.17
Married or living with partner–*N* (%)	366 (24.1)
School attainment > 13 years–*N* (%)	699 (46.0)
*N* COVID19-infected people *per* region–M ± SD	10,776.77 ± 15,870.47
Personal status during lockdown^†^	
Isolation–*N* (%)	1,171 (77.1)
Quarantine–*N* (%)	235 (15.5)
Working during lockdown–*N* (%)	113 (7.4)
Smart-working–*N* (%)	577 (38.0)
*N* of cohabitants during lockdown phase one–M ± SD	2.43 ± 1.39
Diagnosis of COVID-19–*N* (%)	10 (0.7)
Friends or relatives with COVID-19 diagnosis–*N* (%)	209 (13.8)
Smokers–*N* (%)	438 (28.8)
Illegal drugs use during lockdown phase one–*N* (%)	64 (4.2)
IES-R total score–M ± SD	26.63 ± 13.56
IES-R ≥ 24–*N* (%)	827 (54.4)
IES-R ≥ 33–*N* (%)	461 (30.3)
CAGE total score–M ± SD	0.31 ± 0.67
CAGE ≥ 2–*N* (%)	108 (7.1)
BSMAS total score–M ± SD	14.28 ± 4.94
BSMAS ≥ 19–*N* (%)	311 (20.5)
mYFAS 2.0 total score–M ± SD	1.41 ± 2.17
FA Diagnosis–*N* (%)	713 (46.9)
I_7_ impulsiveness total score–M ± SD	6.62 ± 3.86

† *“Phase one,” the phase during which the exponential curve of the contagions is growing*.

*M, mean; SD, standard deviation; BMI, Body Mass Index; COVID-19, Corona Virus Disease 19; IES-R, Impact of Event Scale-Revised; CAGE, self-report measure of alcohol use problems; BSMAS, Bergen Social Media Addiction Scale; mYFAS 2.0, modified Yale Food Addiction Scale Version 2.0*.

COVID-19 related distress was assessed with the 22-items of the Impact of Event Scale-Revised [IES-R; (27)], a widely-used measure investigating the current subjective distress in response to a specific traumatic event. Items scored on a 5-point Likert scale (from 0 = “not at all” to 4 = “extremely”) and assessed the major symptom of post-traumatic stress disorder (PTSD): intrusion, avoidance, and hyper-arousal. The total score ranges from 0 to 88 with higher scores indicating more severe post-traumatic stress symptoms. The instructions of IES-R scale have been specifically tailored for the COVID-19 context (i.e., “phase one” of COVID-19 pandemic, which is considered the traumatic event object of the study). Higher scores indicated more severe stress-related symptoms. We used the Italian adaptation of the IES-R (28), and the Cronbach's α was 0.88. Although there is no specific cut-off score, while scores higher than 23 are considered clinically concerning (29), a total score of 33 represents the best cut-off for a probable diagnosis of PTSD (30).

Alcohol problems were investigated with the CAGE questionnaire, a screening tool composed of 4 dichotomous (1= yes; 0= no) items (31). Total score ranges from 0 to 4 with a higher score reflecting more severe problematic patterns of alcohol use. A cut-off of ≥ 2 is widely used (32) to screen problematic alcohol use (PAU). We used the Italian adaptation of the CAGE (33), and the Cronbach's α was 0.52.

Addiction-like symptoms in relation to excessive and compulsive social media use was assessed through the six-item of the Bergen Social Media Addiction Scale [BSMAS; (34)]. BSMAS items (rated on a 5-point Likert scale, 1 = very rarely to 5= very often) investigate core addiction elements (i.e., salience, mood modification, tolerance, withdrawal, conflict, and relapse) related to social media (Facebook, Instagram, etc.) use in the last 12 months (in this study, items were referred to a time period of the last 2 weeks). Higher BSMAS scores reflect higher SMA symptoms. A cut-off of ≥ 19 is thought to be the ideal threshold identifying individuals at risk of problematic social media use (35). We used the Italian adaptation of the BSMAS (36), and the Cronbach's α was 0.79.

FA was assessed with the modified Yale Food Addiction Scale Version 2.0 [mYFAS 2.0; (37)]. It is composed of 13 items, rated on an 8-point Likert scale (from 0= *never* to 7= *every day*) assessing addictive eating behaviors according to the DSM-5 criteria for substance-related and addictive disorders (37). The mYFAS 2.0. provides two scoring options: a symptom count version (scores ranging from 0 to 11) and a diagnostic version based on the last edition of the DSM criteria (21). We used the Italian adaptation of the mYFAS 2.0 (38), and the Cronbach's α in was 0.89. For this study, items were referred to a time period of the last 2 weeks.

Trait-impulsivity was assessed with the 19 dichotomous (yes/no) items of the impulsiveness subscale of the I_7_ impulsiveness-venturesomeness-empathy scale (39). We used the Italian adaptation of the I_7_ (40, 41), and the Cronbach's α was 0.79.

### Statistical Analyses

All analyses were performed using the SPSS (18.0) statistical package (IBM, Armonk, NY, USA). Hierarchical multiple regression analyses were performed to investigate whether COVID-19 related variables were significant predictors of the different addictive symptoms (i.e., CAGE, BSMAS, and mYFAS total scores), when possible confounding variables were controlled for. The predictors were entered into the regression model according to the following blocks: (1) general data (i.e., gender, age, BMI, educational level, marital status), (2) possible competing predictors (i.e., impulsivity, other addictions), and (3) COVID-19 related variables. We included the following COVID-19 related variables: personal status during lockdown (i.e., isolation, quarantine or neither), diagnosis to COVID-19, friends/relatives with COVID-19 diagnosis, smart working during the lockdown, numbers of infections *per* regions, number of cohabitants during the lockdown, and the IES-R total score. The enter method was used. The associations were reported as standardized beta coefficients (β) and their *p*-values. We also computed zero-order correlations (see Supplementary Table 1) considering *r*= ±0.1 as small, ±0.30 medium, and ±0.50 large effect sizes (42).

## Results

In this sample, during the lockdown, 1,171 (77.1%) of the participants were in isolation and 235 (15.5%) were in quarantine. Moreover, 10 participants (0.7%) received COVID-19 diagnosis and 209 (13.8%) had a relative and/or friend(s) with COVID-19 diagnosis.

According to the IES-R cut-off scores (29, 30), there were 827 (54.4%) participants who met the criteria for clinical-level of stress-related problems and 461 (30.3%) who met the criteria for a probable diagnosis of PTSD. There were 108 participants (7.1%) who met the criteria for PAU, 311 (20.5%) who met the criteria for SMA, and 713 (46.9%) who met the criteria for a diagnosis of FA. Finally, 64 (4.2%) participants reported use of illegal drugs. Clinical and socio-demographic characteristics are reported in Table 1.

### COVID-19 Outbreak and Alcohol Problems

The models explained between 0.003 and 0.07% of the variance (Table 2). In the last block, when controlling for other variables, the IES-R total score remained independently associated with CAGE total score (β = 0.058; *p* = 0.043). Although the model was significant (*F* = 7.850; *p* < 0.001), it did not increase the variance (*R*^2^ *Change* = 0.005; *p* = 0.332). In the last block, male gender (β = 0.090; *p* = 0.001), being a smoker (β = 0.140; *p* < 0.001), higher impulsivity (β = 0.133; *p* < 0.001), and higher FA symptom (β = 0.062; *p* = 0.028) were independently associated with CAGE total score.

**Table 2:** Hierarchical multiple regressions predicting problematic alcohol problems in all the sample (*N* = 1519).

**Dependent variables: CAGE total score**							
	***β***	***p***	**[95% CI]**	**Adjusted** ***R***^**2**^	***F***	***R***^**2**^ **Change**	***F*** **Change**
Block 1 independent variables				0.003	1.868^1^	0.006	1.868
Women	−0.048	0.071	[−0.158; 0.007]				
Age	−0.037	0.243	[−0.006; 0.002]				
BMI	0.020	0.735	[−0.005; 0.012]				
School attainment > 13 years	−0.019	0.472	[−0.095; 0.044]				
Married or living with partner	−0.026	0.399	[−0.135; 0.054]				
Block 2 independent variables				0.071	12.535^2^***^^	0.071^***^	23.065^***^
Women	−0.079	**0.003**	[−0.205; −0.042]				
Age	0.023	0.468	[−0.002; 0.005]				
BMI	−0.004	0.873	[−0.009; 0.008]				
School attainment > 13 years	0.006	0.820	[−0.060; 0.076]				
Married or living with partner	−0.018	0.550	[−0.120;0.064]				
I_7_ impulsiveness total score	0.141	** <0.001**	[0.015;0.034]				
Smokers	0.139	** <0.001**	[0.130; 0.281]				
Illegal drugs use during lockdown^†^	0.037	0.046	[−0.044; 0.292]				
mYFAS 2.0 total score	0.070	**0.012**	[0.005; 0.039]				
BSMAS total score	0.074	**0.010**	[0.002; 0.018]				
Block 3 independent variables				0.071	7.850^3^***^^	0.005	1.145
Women	−0.090	**0.001**	[−0.224; −0.058]				
Age	0.035	0.284	[−0.002; 0.006]				
BMI	−0.003	0.916	[−0.009; 0.008]				
School attainment > 13 years	0.017	0.516	[−0.046; 0.092]				
Married or living with partner	−0.018	0.543	[−0.121; 0.064]				
I_7_ impulsiveness total score	0.133	** <0.001**	[0.014; 0.032]				
Smokers	0.140	** <0.001**	[0.131; 0.283]				
Illegal drugs use during lockdown^†^	0.036	0.164	[−0.049; 0.288]				
mYFAS 2.0 total score	0.062	**0.028**	[0.002; 0.036]				
BSMAS total score	0.055	0.063	[0.000; 0.015]				
Personal status during lockdown^†^	−0.018	0.478	[−0.099; 0.046]				
N of cohabitants during lockdown^†^	0.033	0.200	[−0.008; 0.040]				
N of COVID19-infected people *per* region	0.021	0.424	[0.000; 0.000]				
Smart-working	−0.029	0.261	[−0.111; 0.030]				
Diagnosis of COVID-19	−0.017	0.506	[−0.549; 0.271]				
Friends/relatives with COVID-19	−0.014	0.588	[−0.124; 0.071]				
IES-R total score	0.058	**0.043**	[0.000; 0.006]				

*** *p < 0.001; Degree of freedom: ^1^5:1509, ^2^10;1504, ^3^17;1497; “Phase one” the phase during which the exponential curve of the contagions is growing, β, standardized beta; CI, confidence interval. Bold values indicate significant variable*.

*CAGE, self-report measure of alcohol use problems; BMI, Body Mass Index; mYFAS 2.0, modified Yale Food Addiction Scale Version 2.0; BSMAS, Bergen Social Media Addiction Scale; COVID-19, Corona Virus Disease 19; IES-R, Impact of Event Scale-Revised*.

### COVID-19 Outbreak and SMA Symptoms

The models explained between 14 and 29% of the variance (Table 3).

**Table 3:** Hierarchical multiple regressions predicting social media addiction symptoms in all the sample (*N* = 1519).

**Dependent variables: BSMAS total score**							
	***β***	***p***	**[95% CI]**	**Adjusted** ***R***^**2**^	***F***	***R***^**2**^ **Change**	***F*** **Change**
Block 1 independent variables				0.138	49.309^1^***^^	0.140^***^	49.309^***^
Women	0.154	** <0.001**	[1.218; 2.341]				
Age	−0.240	** <0.001**	[−0.135; −0.082]				
BMI	0.013	0.593	[−0.042; 0.074]				
School attainment > 13 years	−0.090	** <0.001**	[−1.372; −0.416]				
Married or living with partner	−0.091	**0.002**	[−1.694; −0.396]				
Block 2 independent variables				0.236	47.841^2^***^^	0.101^***^	40.001^***^
Women	0.107	** <0.001**	[0.693; 1.777]				
Age	−0.191	** <0.001**	[−0.112; −0.061]				
BMI	−0.060	**0.015**	[−0.128; −0.014]				
School attainment > 13 years	−0.059	**0.011**	[−1.038; −0.133]				
Married or living with partner	−0.086	**0.002**	[−1.601; −0.378]				
I_7_ impulsiveness total score	0.179	** <0.001**	[0.169; 0.290]				
Smokers	−0.078	**0.001**	[−1.359; −0.344]				
Illegal drugs use during lockdown^†^	−0.018	0.430	[−1.575; 0.671]				
mYFAS 2.0 total score	0.228	** <0.001**	[0.409; 0.629]				
CAGE total score	0.060	**0.010**	[0.108; 0.782]				
Block 3 independent variables				0.292	37.724^3^***^^	0.059^***^	17.897^***^
Women	0.055	**0.019**	[0.104; 1.172]				
Age	−0.155	** <0.001**	[−0.095; −0.046]				
BMI	−0.048	**0.045**	[−0.112; −0.001]				
School attainment > 13 years	−0.039	0.087	[−0.834; 0.057]				
Married or living with partner	−0.076	**0.003**	[−1.473; −0.291]				
I_7_ impulsiveness total score	0.133	** <0.001**	[0.111; 0.229]				
Smokers	−0.073	**0.001**	[−1.289; −0.306]				
Illegal drugs use during lockdown^†^	−0.028	0.221	[−1.759; 0.407]				
mYFAS 2.0 total score	0.173	** <0.001**	[0.285; 0.502]				
CAGE total score	0.042	0.063	[−0.017; 0.635]				
Personal status during lockdown^†^	−0.068	**0.003**	[−0.178; −0.249]				
N of cohabitants during lockdown^†^	0.006	0.791	[−0.135; 0.178]				
N of COVID19-infected people *per* regions	0.008	0.714	[0.000; 0.000]				
Smart-working	−0.028	0.212	[−0.740; 0.164]				
Diagnosis of COVID-19	0.002	0.944	[−2.541; 2.731]				
Friends/relatives with COVID-19	−0.005	0.826	[−0.697; 0.557]				
IES-R total score	0.259	** <0.001**	[0.077; 0.111]				

*** *p < 0.001; Degree of freedom: ^1^5:1509, ^2^10;1504, ^3^17;1497; “Phase one” the phase during which the exponential curve of the contagions is growing; β, standardized beta; CI, confidence interval. Bold values indicate significant variable*.

*BSMAS, Bergen Social Media Addiction Scale; BMI, Body Mass Index; mYFAS 2.0, modified Yale Food Addiction Scale Version 2.0; CAGE, self-report measure of alcohol use problems; COVID-19, Corona Virus Disease 19; IES-R, Impact of Event Scale-Revised*.

The last block, which included COVID-19 related variables, increased significantly the variance (*R*^2^ *Change* = 0.059; *p* < 0.001), and when controlling for other variables, the IES-R total score (β = 0.259; *p* < 0.001) was independently associated with BSMAS total score. A more severe self-reported COVID-19 related distress was associated with more SMA symptoms. Personal status during lockdown (i.e., being in quarantine/isolation) was also independently associated with BSMAS total score (β = −0.061; *p* = 0.018). Female gender (β = 0.055; *p* = 0.019), age (β = −0.155; *p* < 0.001), being unmarried (β = 0.076; *p* = 0.003), and a smoker (β = −0.073; *p* = 0.001), higher impulsivity (β = 0.133; *p* < 0.001) and higher FA symptoms (β = 0.173; *p* < 0.001) were also independently associated with BSMAS total score in the last block.

### COVID-19 Outbreak and FA Symptoms

The models explained between 12 and 22% of the variance (Table 4).

**Table 4:** Hierarchical multiple regressions predicting food addiction symptoms in all the sample (*N* = 1519).

**Dependent variables: mYFAS 2.0 total score**							
	***β***	***p***	**[95% CI]**	**Adjusted** ***R***^**2**^	***F***	***R***^**2**^ **Change**	***F*** **Change**
Block 1 independent variables				0.118	41.386^1^***^^	0.221^***^	41.386^***^
Women	0.186	** <0.001**	[0.695; 1.194]				
Age	−0.134	** <0.001**	[−0.039; −0.015]				
BMI	0.296	** <0.001**	[0.129; 0.180]				
School attainment > 13 years	−0.062	**0.012**	[−0.484; −0.059]				
Married or living with partner	−0.037	0.204	[−0.475; 0.101]				
Block 2 independent variables				0.208	40.847^2^***^^	0.093^***^	35.568^***^
Women	0.148	** <0.001**	[0.511; 0.993				
Age	−0.052	0.074	[−0.022; 0.001]				
BMI	0.290	** <0.001**	[0.127; 0.176]				
School attainment > 13 years	−0.028	0.235	[−0.326; 0.080]				
Married or living with partner	−0.016	0.550	[−0.358; 0.191]				
I_7_ impulsiveness total score	0.129	** <0.001**	[0.045; 0.100]				
Smokers	0.043	0.077	[−0.022; 0.433]				
Illegal drugs use during lockdown^†^	0.026	0.266	[−0.218; 0.787]				
CAGE total score	0.060	**0.012**	[0.043;0.344]				
BSMAS total score	0.236	** <0.001**	[0.082; 0.126]				
Block 3 independent variables				0.223	26.593^3^***^^	0.018^**^	5.114^***^
Women	0.122	**0.001**	[0.373; 0.862]				
Age	−0.049	0.100	[−0.021; 0.002]				
BMI	0.285	** <0.001**	[0.124; 0.173]				
School attainment > 13 years	−0.024	0.320	[−0.309; 0.101]				
Married or living with partner	−0.014	0.601	[−0.346; 0.200]				
I_7_ impulsiveness total score	0.109	** <0.001**	[0.034; 0.088]				
Smokers	0.039	0.106	[−0.004; 0.414]				
Illegal drugs use during lockdown	0.020	0.399	[−0.284; 0.713]				
CAGE total score	0.052	**0.028**	[0.018; 0.317]				
BSMAS total score	0.190	** <0.001**	[0.060; 0.106]				
Personal status during lockdown^†^	−0.009	0.693	[−0.171; 0.258]				
N of cohabitants during lockdown^†^	−0.010	0.681	[−0.087; 0.057]				
N of COVID19-infected people *per* regions	0.004	0.881	[0.000;0.000]				
Smart-working	−0.013	0.584	[−0.266; 0.150]				
Diagnosis of COVID-19	0.019	0.406	[−0.699; 1.727]				
Friends/relatives with COVID-19	−0.002	0.918	[−0.304; 0.273]				
IES-R total score	0.150	** <0.001**	[0.016; 0.032]				

** p < 0.01;

*** *p < 0.001; Degree of freedom: ^1^5:1509, ^2^10;1504, ^3^17;1497; “Phase one” the phase during which the exponential curve of the contagions is growing; β, standardized beta; CI, confidence interval. Bold values indicate significant variable*.

*mYFAS 2.0, modified Yale Food Addiction Scale Version 2.0; BMI, Body Mass Index; CAGE, self-report measure of alcohol use problems; BSMAS, Bergen Social Media Addiction Scale; COVID-19, Corona Virus Disease 19; IES-R, Impact of Event Scale-Revised*.

The last block, which included COVID-19 related variables, increased significantly such a variance (*R*^2^ *Change* = 0.018; *p* < 0.001), and when controlling for other variables, the IES-R total score (β = 0.150; *p* < 0.001) was independently associated with mYFAS 2.0 total score. A more severe self-reported COVID-19 related distress was associated with more FA symptoms. Female gender (β = 0.122; *p* < 0.001), higher BMI (β = 0.285; *p* < 0.001), impulsivity (β = 0.109; *p* < 0.001), SMA symptoms (β = 0.190; *p* < 0.001), and alcohol problems (β = 0.052; *p* = 0.028) were also independently associated with mYFAS 2.0 total score in the last block.

## Discussion

The main aim of this study was to investigate the association between COVID-19 related distress and addictive symptoms (i.e., alcohol problems, SMA and FA) during “phase one” of the Italian lockdown (9th March 2020–4th May 2020). In line with previous reports on the psychological impact of quarantine (4, 43) during the lockdown, in the current sample, 54.4% of the participants self-reported a significant psychological impact of COVID-19, as assessed by the IES-R (27).

Our results seem to confirm that one of the most negative impacts of the COVID-19 emergency could be related to an increase in the prevalence and severity of both substance and behavioral addictions (11–15, 44, 45). Our data showed that the self-reported psychological impact of the COVID-19 was positively correlated (Supplementary Table 1) with alcohol problems (small effect size), SMA symptoms (medium to large effect size) and FA symptoms (medium effect size). At a multivariate level, when controlling for potential confounding variables that have been traditionally related to addictive disorders [e.g., impulsivity (46–48)], the IES-R remained independently associated with CAGE, BSMAS, and mYFAS 2.0 total scores. However, neither self-reported COVID-19 related distress, nor the other variables related to this emergency (e.g., isolation/quarantine) were significantly associated with increased CAGE total score variability. This result seems to be in accordance with the scenario supposed by Rehm et al. (13) regarding the consumption of alcohol during the COVID-19 emergency. According to a literature search focused on the impacts of past public health and economic crises on alcohol consumption, the authors hypothesized a decrease in alcohol consumption in the immediate future, followed by an increase in the medium- and longer-term future (13). Although solitary drinking among young adults appears to be associated with drinking problems (49), it is also known that the social context plays a crucial role in PAU (50). It is possible that during lockdown people were more prone to cope with their negative feelings through use of social media and consumption of high calorie food. Accordingly, it should be noted that during the “stay-at home” ordinance, engaging with social networks was the only possible way to communicate with others. Although keeping social contact remotely with people reduces the psychological impacts of isolation, the excessive engagement with technology is associated with several risks, especially when used to reduce stress (15). Indeed, despite any temporary and immediate gratifying effects derived from social networking, long-term effects are potentially addictive (51) and are associated with several negative outcomes including emotional and relational problems (52).

Our results showed that COVID-19 related distress was also associated with higher FA symptoms. Similar to other rewarding stimuli, compulsive and uncontrolled overeating could reflect a dysfunctional coping strategy consisting of “comfort food” used to escape from an unpleasant state and/or to self-regulate emotions (19, 53). From a neurophysiological point of view, it has been suggested that the natural reward of highly palatable food can reduce the activity of the Hypothalamic-Pituitary-Adrenal axis and the production of cortisol (54–56). The constant repetition of this pattern could lead to neurobehavioral adaptations promoting FA (54–56).

The current study extends previous research and could provide useful information to be taken into account when lockdowns were implemented. To the best of our knowledge, this is the first study shedding light on the relationship between COVID-19 related distress and different types of addictive symptoms. This research focuses on a specific class of symptoms that could give rise to addiction disorders through a “subtle way.” Indeed, such disorders do not entail the use of illegal substances and are easily accessible during the lockdown. Research evidence concerning such phenomena would seem to be extendable to situations where traumatic events do not occur on a global scale (57), but at an individual level (58). Furthermore, the survey is based on an adequate sample size, across all regions of a country that has been strongly affected by COVID-19 infection, and the psychological pressure due to the spread of the infection has been considered in the statistical analyses. Moreover, Italy ran into the outbreak before other countries, thus it offers a relevant scenario concerning public health issues that could be very informative for other countries around the globe.

Nonetheless, some limitations of the study need to be acknowledged. For instance, these findings cannot be extended to adolescent populations. Moreover, this is a cross-sectional study and it is difficult to draw causal conclusions. Furthermore, although online surveys have remarkable advantages (e.g., access to unique populations, such as individuals in isolation/quarantine), there are also disadvantages, such as the selection bias, that should be considered (59). For example, notwithstanding increasing Internet use and availability in the society at large, it is known (60) that online questionnaires might be more accessible to some groups of individuals (e.g., students) compared to others (e.g., frail elders, the poorest). Similarly, and accordingly with the present data, it has been reported (61) that online surveys response rate might be biased in favor of female' participants, probably because of gender differences in online behaviors (e.g., women make intense use of social networks, whereas men are more engaged in online games) (62–64). Lastly, it should be noted that, although the CAGE is widely used to screen PAU (32), in the present sample a low Cronbach's α (i.e., 0.52) was detected. A review on 22 studies (65) showed that CAGE reliability coefficients ranged from 0.52 to 0.90, indicating considerable variability of this self-report, which seems to be affected by sample age (i.e., older CAGE respondents generally producing more reliable scores than younger ones). Thus, future reports should investigate the association between COVID-19 related distress and PAU using alternate alcohol screening tool such as the Alcohol Use Disorders Identification Test [AUDIT; (66)].

Future studies might also highlight protective factors that clinicians should take into account during outbreaks to reduce the side-effects of restrictions. Based on the results of this research, policy-makers need to address issues related to the COVID-19 pandemic in two ways: (i) media campaigns for health promotion aimed at increasing people's awareness about the risk of developing “subtle addictions,” that do not entail the intake of illegal substances, (ii) tailoring *ad-hoc* on-line interventions during the lockdown and face-to-face clinical interventions after such a phase, to avoid these symptoms giving rise to pathological disorders. An overarching message of this work consists in highlighting the need to take into account addictive symptoms at three levels: (i) when scholars design researches studies investigating outbreak-related phenomena, (ii) when clinicians carry out outbreak-related interventions, and (iii) when policy-makers make public health decisions. For instance, epidemiological studies should monitor the incidence of such addiction symptoms to provide timely information. Monitoring such a phenomenon might implement policies at national level to cope with the incidence of these addictions in society at large, given that subsequent economic and social costs might be higher for the welfare system.

Lastly, our results suggest the need to implement applied psychological strategies aimed at helping people to cope with addictive behaviors during lockdown conditions. These strategies could be included in most of the extant psychological intervention protocols developed to face COVID-19 emergency (67) and/or could be adapted according to current evidence-based programs, such as the Screening, Brief Intervention, and Referral to Treatment [SBIRT; (68)].

To conclude, our results seem to confirm the concerns (11–15) about the negative impact of the COVID-19 emergency on addictive behaviors, suggesting that this issue should be carefully monitored when social distancing occurs. The coronavirus disease 2019 pandemic is a global health crisis requiring, on the one hand, clinicians to be prepared to cope with the increase in the psychopathological symptoms incidence, including those related to addictive behavior. On the other hand, scholars have to design studies and provide guidelines to cope with such crises. Finally, policy-makers should take into account scholars' information to support and stimulate clinical interventions when addressing public health issues related to pandemic emergencies.

## Data Availability Statement

The raw data supporting the conclusions of this article will be made available by the authors, without undue reservation.

## Ethics Statement

This research was approved by the ethics committee of the European University of Rome (Prot. N.004/20) in line with the Helsinki declaration standards. The patients/participants provided their written informed consent to participate in this study.

## Author Contributions

AP: conceptualization, supervision, methodology, data curation, software and formal analysis, and writing—original draft preparation. GC: conceptualization, methodology, data curation, and writing—review and editing. CM: conceptualization, methodology, data curation, and writing—review and editing. BF: methodology, supervision, and writing—review and editing. CI: conceptualization, supervision, methodology, data curation, software and formal analysis, and writing—original draft preparation. All authors: contributed to the article and approved the submitted version.

## Conflict of Interest

The authors declare that the research was conducted in the absence of any commercial or financial relationships that could be construed as a potential conflict of interest.

## References

[1] Johns Hopkins Coronavirus Resource Center Available online at: https://coronavirus.jhu.edu/map.html (2020) (accessed November 05, 2020).

[2] Li Z Ge J Yang M Feng J Qiao M Jiang R. Vicarious traumatization in the general public, members, and non-members of medical teams aiding in COVID-19 control. Brain Behav Immun. (2020) 88:916–9. 10.1101/2020.02.29.20029322 32169498 PMC7102670

[3] Chen Q Liang M Li Y Guo J Fei D Wang L. Mental health care for medical staff in China during the COVID-19 outbreak. Lancet Psychiatry. (2020) 7:e15–6. 10.1016/S2215-0366(20)30078-X 32085839 PMC7129426

[4] Brooks SK Webster RK Smith LE Woodland L Wessely S Greenberg N. The psychological impact of quarantine and how to reduce it: rapid review of the evidence. Lancet. (2020) 395:912–20. 10.1016/S0140-6736(20)30460-8 32112714 PMC7158942

[5] Stefana A Youngstrom EA Hopwood C Dakanalis A. The pandemic brings a second wave of social isolation and disrupted services. Eur Arch Psychiatry Clin Neurosci. (2020) 270:785–6. 10.1007/s00406-020-01137-8 32415510 PMC7227800

[6] Pappa S Ntella V Giannakas T Giannakoulis VG Papoutsi E Katsaounou P. Prevalence of depression, anxiety, and insomnia among healthcare workers during the COVID-19 pandemic: a systematic review and meta-analysis. Brain Behav Immun. (2020) 88:901–7. 10.2139/ssrn.3594632 32437915 PMC7206431

[7] Hiremath P Suhas Kowshik CS Manjunath M Shettar M. COVID 19: impact of lock-down on mental health and tips to overcome. Asian J Psychiatr. (2020) 51:102088. 10.1016/j.ajp.2020.102088 32302964 PMC7151434

[8] Mukhtar S. Psychological health during the coronavirus disease 2019 pandemic outbreak. Int J Soc Psychiatry. (2020) 66:512–6. 10.1177/0020764020925835 32434402 PMC7405632

[9] Mackolil J. Addressing psychosocial problems associated with the COVID-19 lockdown. Asian J Psychiatr. (2020) 51:102156. 10.1016/j.ajp.2020.102156 32413617 PMC7207101

[10] Torjesen I. Covid-19: mental health services must be boosted to deal with “tsunami” of cases after lockdown. BMJ. (2020) 369:m1994. 10.1136/bmj.m1994 32417756

[11] Lippi G Henry BM Bovo C Sanchis-Gomar F. Health risks and potential remedies during prolonged lockdowns for coronavirus disease 2019 (COVID-19). Diagnosis (Berl). (2020) 7:85–90. 10.1515/dx-2020-0041 32267243

[12] Volkow ND. Collision of the COVID-19 and addiction epidemics. Ann Intern Med. (2020) 173:61–2. 10.7326/M20-1212 32240293 PMC7138334

[13] Rehm J Kilian C Ferreira-Borges C Jernigan D Monteiro M Parry CDH. Alcohol use in times of the COVID 19: implications for monitoring and policy. Drug Alcohol Rev. (2020) 39:301–4. 10.1111/dar.13074 32358884 PMC7267161

[14] Marsden J Darke S Hall W Hickman M Holmes J Humphreys K. Mitigating and learning from the impact of COVID-19 infection on addictive disorders. Addiction. (2020) 115:1007–10. 10.1111/add.15080 32250482 PMC9364227

[15] Kiraly O Potenza MN Stein DJ King DL Hodgins DC Saunders JB. Preventing problematic internet use during the COVID-19 pandemic: consensus guidance. Compr Psychiatry. (2020) 100:152180. 10.1016/j.comppsych.2020.152180 32422427 PMC7215166

[16] Pearl RL. Weight Stigma and the “Quarantine-15”. Obesity (Silver Spring). (2020). 10.1002/oby.22850 32324954 PMC7264559

[17] Gordon EL Lent MR Merlo LJ. The effect of food composition and behavior on neurobiological response to food: a review of recent research. Curr Nutr Rep. (2020) 9:75–82. 10.1007/s13668-020-00305-5 32157660

[18] Gordon EL Ariel-Donges AH Bauman V Merlo LJ. What is the evidence for “food addiction?” A systematic review. Nutrients. (2018) 10:477. 10.3390/nu10040477 29649120 PMC5946262

[19] Imperatori C Fabbricatore M Vumbaca V Innamorati M Contardi A Farina B. Food addiction: definition, measurement and prevalence in healthy subjects and in patients with eating disorders. Riv Psichiatr. (2016) 51:60–5. 10.1708/2246.24196 27183510

[20] Meule A Gearhardt AN. Food addiction in the light of DSM-5. Nutrients. (2014) 6:3653–71. 10.3390/nu6093653 25230209 PMC4179181

[21] American Psychiatric Association Diagnostic and Statistical Manual of Mental Disorders - DSM-5. 5th ed Arlington, VA: American Psychiatric Publishing (2013). 10.1176/appi.books.9780890425596

[22] Kalon E Hong JY Tobin C Schulte T. Psychological and neurobiological correlates of food addiction. Int Rev Neurobiol. (2016) 129:85–110. 10.1016/bs.irn.2016.06.003 27503449 PMC5608024

[23] Li Y Wang Y Jiang J Valdimarsdottir UA Fall K Fang F Psychological distress among health professional students during the COVID-19 outbreak. Psychol Med. (2020) 1–3. 10.1017/S0033291720001555 PMC7225209 32389148

[24] Faul F Erdfelder E Buchner A Lang AG. Statistical power analyses using G^*^Power 3.1: tests for correlation and regression analyses. Behav Res Methods. (2009) 41:1149–60. 10.3758/BRM.41.4.1149 19897823

[25] Barberio AM Alareeki A Viner B Pader J Vena JE Arora P. Central body fatness is a stronger predictor of cancer risk than overall body size. Nat Commun. (2019) 10:383. 10.1038/s41467-018-08159-w 30670692 PMC6342989

[26] Italian Ministry of Health (2020) Available online at: http://www.salute.gov.it/portale/nuovocoronavirus/dettaglioContenutiNuovoCoronavirus.jsp?lingua=italiano&id=5351&area=nuovoCoronavirus&menu=vuoto

[27] Weiss DS Marmar CR. The impact of event scale–revised. In: Wilson JP Keane TM, editors. Assessing Psychological Trauma and PTSD. New York, NY: Guilford Press (1997). p. 399–411. 10.1037/t12199-000

[28] Craparo G Faraci P Rotondo G Gori A. The Impact of Event Scale - Revised: psychometric properties of the Italian version in a sample of flood victims. Neuropsychiatr Dis Treat. (2013) 9:1427–32. 10.2147/NDT.S51793 24092980 PMC3788700

[29] Asukai N Kato H Kawamura N Kim Y Yamamoto K Kishimoto J. Reliability and validity of the Japanese-language version of the impact of event scale-revised (IES-R-J): four studies of different traumatic events. J Nerv Ment Dis. (2002) 190:175–82. 10.1097/00005053-200203000-00006 11923652

[30] Creamer M Bell R Failla S. Psychometric properties of the impact of event scale - revised. Behav Res Ther. (2003) 41:1489–96. 10.1016/j.brat.2003.07.010 14705607

[31] Ewing JA. Detecting alcoholism: the CAGE questionnaire. Jama. (1984) 252:1905–7. 10.1001/jama.252.14.1905 6471323

[32] Dhalla S Kopec JA. The CAGE questionnaire for alcohol misuse: a review of reliability and validity studies. Clin Invest Med. (2007) 30:33–41. 10.25011/cim.v30i1.447 17716538

[33] Agabio R Marras P Gessa GL Carpiniello B. Alcohol use disorders, and at-risk drinking in patients affected by a mood disorder, in Cagliari, Italy: sensitivity and specificity of different questionnaires. Alcohol Alcoholism. (2007) 42:575–81. 10.1093/alcalc/agm072 17766313

[34] Schou Andreassen C Billieux J Griffiths MD Kuss DJ Demetrovics Z Mazzoni E. The relationship between addictive use of social media and video games and symptoms of psychiatric disorders: a large-scale cross-sectional study. Psychol Addict Behav. (2016) 30:252–62. 10.1037/adb0000160 26999354

[35] Banyai F Zsila A Kiraly O Maraz A Elekes Z Griffiths MD. Problematic social media use: results from a large-scale nationally representative adolescent sample. PLoS ONE. (2017) 12:e0169839. 10.1371/journal.pone.0169839 28068404 PMC5222338

[36] Monacis L de Palo V Griffiths MD Sinatra M. Social networking addiction, attachment style, and validation of the Italian version of the Bergen Social Media Addiction Scale. J Behav Addict. (2017) 6:178–86. 10.1556/2006.6.2017.023 28494648 PMC5520120

[37] Schulte EM Gearhardt AN. Development of the modified yale food addiction scale version 2.0. Eur Eat Disord Rev. (2017) 25:302–8. 10.1002/erv.2515 28370722

[38] Imperatori C Fabbricatore M Lester D Manzoni GM Castelnuovo G Raimondi G. Psychometric properties of the modified Yale Food Addiction Scale Version 2.0 in an Italian non-clinical sample. Eat Weight Disord. (2019) 24:37–45. 10.1007/s40519-018-0607-x 30414076

[39] Eysenck SB Pearson PR Easting G Allsopp JF Age norms for impulsiveness, venturesomeness and empathy in adults. Pers Individ Diff . (1985) 6:613–9. 10.1016/0191-8869(85)90011-X

[40] Russo PM Leone L De Pascalis V. Cross-cultural validity of the I7 impulsiveness-venturesomeness-empathy scales: evidence from the Italian I7. Compr Psychiatry. (2011) 52:446–52. 10.1016/j.comppsych.2010.07.008 21683182

[41] Panno A Sarrionandia A Lauriola M Giacomantonio M. Alexithymia and risk preferences: predicting risk behaviour across decision domains. Int J Psychol. (2019) 54:468–77. 10.1002/ijop.12479 29460281

[42] Cohen J. Statistical Power Analysis for the Behavioral Sciences (2nd ed.). Hillsdale: Erlbaum (1988).

[43] Cellini N Canale N Mioni G Costa S. Changes in sleep pattern, sense of time and digital media use during COVID-19 lockdown in Italy. J Sleep Res. (2020) 2020:e13074. 10.31234/osf.io/284mr 32410272 PMC7235482

[44] Rolland B Haesebaert F Zante E Benyamina A Haesebaert J Franck N. Global changes and factors of increase in caloric/salty food, screen, and substance use, during the early COVID-19 containment phase in France: a general population online survey. JMIR Public Health Surveill. (2020) 6:e19630. 10.2196/preprints.19630 32589149 PMC7505683

[45] Sun Y Li Y Bao Y Meng S Schumann G Kosten T. Brief report: increased addictive internet and substance use behavior during the COVID-19 pandemic in China. Am J Addict. (2020) 29:268–70. 10.1111/ajad.13066 32500608 PMC7300868

[46] Maxwell AL Gardiner E Loxton NJ. Investigating the relationship between reward sensitivity, impulsivity, and food addiction: a systematic review. Eur Eat Disord Rev. (2020) 28:368–84. 10.1002/erv.2732 32142199

[47] Dalley JW Ersche KD. Neural circuitry and mechanisms of waiting impulsivity: relevance to addiction. Philos Trans R Soc Lond B Biol Sci. (2019) 374:20180145. 10.1098/rstb.2018.0145 30966923 PMC6335458

[48] Lee RSC Hoppenbrouwers S Franken I. A systematic meta-review of impulsivity and compulsivity in addictive behaviors. Neuropsychol Rev. (2019) 29:14–26. 10.1007/s11065-019-09402-x 30927147

[49] Skrzynski CJ Creswell KG. Associations between solitary drinking and increased alcohol consumption, alcohol problems, and drinking to cope motives in adolescents and young adults: a systematic review and meta-analysis. Addiction. (2020) 115:1989–2007. 10.1111/add.15055 32196794 PMC8053066

[50] Hendriks H Van den Putte B Gebhardt WA Moreno MA. Social drinking on social media: content analysis of the social aspects of alcohol-related posts on Facebook and Instagram. J Med Internet Res. (2018) 20:e226. 10.2196/jmir.9355 29934290 PMC6035352

[51] Hormes JM Kearns B Timko CA. Craving Facebook? Behavioral addiction to online social networking and its association with emotion regulation deficits. Addiction. (2014) 109:2079–88. 10.1111/add.12713 25170590

[52] Andreassen CS Online social network site addiction: a comprehensive review. Curr Addict Rep. (2015) 2:175–84. 10.1007/s40429-015-0056-9

[53] Panno A Lauriola M Pierro A. Regulatory mode and risk-taking: the mediating role of anticipated regret. PLoS ONE. (2015) 10:e0143147. 10.1371/journal.pone.0143147 26580960 PMC4651368

[54] Ulrich-Lai YM Fulton S Wilson M Petrovich G Rinaman L. Stress exposure, food intake and emotional state. Stress. (2015) 18:381–99. 10.3109/10253890.2015.1062981 26303312 PMC4843770

[55] Yau YH Potenza MN. Stress and eating behaviors. Minerva Endocrinol. (2013) 38:255–67. 24126546 PMC4214609

[56] Adam TC Epel ES. Stress, eating and the reward system. Physiol Behav. (2007) 91:449–58. 10.1016/j.physbeh.2007.04.011 17543357

[57] DiMaggio C Galea S Li G. Substance use and misuse in the aftermath of terrorism. A Bayesian meta-analysis. Addiction. (2009) 104:894–904. 10.1111/j.1360-0443.2009.02526.x 19392912

[58] Teixeira CAB Lasiuk G Barton S Fernandes MNF Gherardi-Donato E. An exploration of addiction in adults experiencing early-life stress: a metasynthesis. Rev Lat Am Enfermagem. (2017) 25:e2939. 10.1590/1518-8345.2026.2939 29020127 PMC5635699

[59] Wright KB Researching Internet-based populations: advantages and disadvantages of online survey research, online questionnaire authoring software packages, and web survey services. J Comput-Mediat Commun. (2005) 10:JCMC1034 10.1111/j.1083-6101.2005.tb00259.x

[60] Remillard ML Mazor KM Cutrona SL Gurwitz JH Tjia J. Systematic review of the use of online questionnaires of older adults. J Am Geriatr Soc. (2014) 62:696–705. 10.1111/jgs.12747 24635138 PMC4098903

[61] Van Mol C Improving web survey efficiency: the impact of an extra reminder and reminder content on web survey response. Int J Sci Res. (2017) 20:317–27. 10.1080/13645579.2016.1185255

[62] Muscanell NL Guadagno RE Make new friends or keep the old: gender and personality differences in social networking use. Comput Hum Behav. (2012) 28:107–12. 10.1016/j.chb.2011.08.016

[63] Kimbrough AM Guadagno RE Muscanell NL Dill J Gender differences in mediated communication: women connect more than do men. Comput Hum Behav. (2013) 29:896–900. 10.1016/j.chb.2012.12.005

[64] Dufour M Brunelle N Tremblay J Leclerc D Cousineau MM Khazaal Y. Gender difference in internet use and internet problems among quebec high school students. Can J Psychiatry. (2016) 61:663–8. 10.1177/0706743716640755 27310231 PMC5348090

[65] Shields AL Caruso JC A reliability induction and reliability generalization study of the CAGE questionnaire. Educ Psychol Meas. (2004) 64:254–70. 10.1177/0013164403261814

[66] Saunders JB Aasland OG Babor TF de la Fuente JR Grant M. Development of the Alcohol Use Disorders Identification Test (AUDIT): WHO collaborative project on early detection of persons with harmful alcohol consumption–II. Addiction. (1993) 88:791–804. 10.1111/j.1360-0443.1993.tb02093.x 8329970

[67] Imperatori C Dakanalis A Farina B Pallavicini F Colmegna F Mantovani F. Global storm of stress-related psychopathological symptoms: a brief overview on the usefulness of virtual reality in facing the mental health impact of COVID-19. Cyberpsychol Behav Soc Netw. (2020). 10.1089/cyber.2020.0339. [Epub ahead of print]. 32640852

[68] Babor TF McRee BG Kassebaum PA Grimaldi PL Ahmed K Bray J. Screening, Brief Intervention, and Referral to Treatment (SBIRT): toward a public health approach to the management of substance abuse. Subst Abus. (2007) 28:7–30. 10.1300/J465v28n03_03 18077300

