# Supplementary material for: COVID-19 Related Distress Is Associated With Alcohol Problems, Social Media and Food Addiction Symptoms: Insights From the Italian Experience During the Lockdown
Source: Front Psychiatry. 2020 Nov 25;11:577135. doi: 10.3389/fpsyt.2020.577135 (PMC7723899; doi:10.3389/fpsyt.2020.577135)
Supplement: Supplementary file 1 [file Table_1.pdf]

Supplementary Table 1. *Intercorrelations among variables investigated in the Study.*

	1	2	3	4	5	6	7
1 – IES-R Total score	–						
2 – CAGE Total score	0.123***	–					
3 – BSMAS Total score	0.407***	0.118***	–				
4 – mYFAS 2.0 Total score	0.295***	0.127***	-0.311***	–			
5 – I_7 impulsiveness Total score	0.260***	0.199***	0.274***	0.235***	–		
6 – Age	-0.204***	-0.045	-0.328***	-0.143***	-0.143***	–	
7 – self-reported BMI	-0.046	-0.023	-0.071**	0.227***	-0.007	0.170***	–

Note: ***p* < 0.01;****p* < 0.001
Abbreviation:
IES-R= Impact of Event Scale – Revised; CAGE = self-report measure of alcohol use problems; BSMAS= Bergen Social Media Addiction Scale; mYFAS 2.0= modified Yale Food Addiction Scale Version 2.0; BMI= Body Mass Index;
